# Peripheral cytokine distinguish recent from *Mycobacterium tuberculosis* infection and active tuberculosis

**DOI:** 10.3389/fmicb.2026.1865147

**Published:** 2026-08-05

**Authors:** Peng Lu, Zhongqi Li, Xiaoyan Ding, Jingjing Pan, Hui Ding, Hanli Yao, Limei Zhu, Lei Huang, Qiao Liu

**Affiliations:** 1 Department of Chronic Communicable Disease, Jiangsu Provincial Center for Disease Control and Prevention, Nanjing, China; 2 Anhui Province Clinical Research Center for Critical Respiratory Medicine, The First Affiliated Hospital of Wannan Medical University (Yijishan Hospital of Wannan Medical College), Wuhu, China; 3 Department of Public Health, Changzhou Third People's Hospital, Zhouchang, China

**Keywords:** biomarker, cytokines, *mycobacterium* infection, recent infection, tuberculosis

## Abstract

**Background:**

Recent *Mycobacterium tuberculosis* infection (*MTB*) carries a higher risk of progression to active tuberculosis (TB) than *MTB* infection, but the immune features that distinguish these stages remain unclear.

**Methods:**

In this multicenter observational study in Jiangsu Province, China, we enrolled 228 participants with *MTB* infection (*n* = 88), recent infection (*n* = 44), or active TB (*n* = 96). Twelve plasma cytokines and chemokines were quantified using a multiplex Luminex platform. Group differences were assessed using non-parametric tests with a two-stage false discovery rate (FDR) correction and age- and sex-adjusted residual models. Biomarker performance was evaluated by repeated Monte Carlo cross-validation using an ensemble feature-selection framework. *Post-hoc* power analyses were conducted to evaluate the robustness of subgroup comparisons.

**Results:**

Age, but not sex, differed across groups. After age- and sex-adjusted comparisons, six markers differed across groups. While recent infection exhibited a trend toward higher IL-23 and IL-12 concentrations compared to remote infection, *post-hoc* analysis revealed these subgroup differences had small effect sizes and were statistically underpowered. In the combined infection-vs.-TB comparison, IL-8 and CCL1 were higher in active TB, whereas IL-23, IL-12, IL-10, and IL-1ra were lower. Among multivariable models, XGBoost performed best (mean AUC 0.866), followed by the ensemble model (0.865) and random forest (0.861). Decision curve analysis suggested potential clinical utility across exploratory threshold probabilities, although no clinically validated operating point was predefined.

**Conclusion:**

Active TB exhibits a distinct peripheral cytokine pattern compared to *MTB* infection. Recent infection may represent an exploratory trend toward preserved Th1/Th17 signaling, though larger longitudinal cohorts are required for confirmation. Peripheral cytokine profiles integrated into multivariable models may provide complementary triage information, although external validation is required before clinical application.

## Introduction

Tuberculosis (TB) remains one of the most consequential infectious diseases worldwide. In 2024, an estimated 10.8 million people developed active TB and approximately 1.25 million died of the disease, underscoring the persistent gap between infection control and disease prevention (Organization. WH, 2025). The global TB strategy has therefore increasingly emphasized earlier identification of infection, targeted prevention, and risk-stratified intervention rather than relying solely on passive case detection (Uplekar et al., 2015).

A fundamental challenge is that *Mycobacterium tuberculosis* (*MTB*) infection is not a binary event. Most exposed individuals do not develop immediate disease, yet a substantial proportion enter a persistent infection state that may remain clinically silent for years. Importantly, the probability of progression is not constant over time: it is highest soon after exposure and then declines, suggesting that recent and *MTB* infection are biologically different states rather than simply different points on the same continuum (Hamada et al., 2023). In practice, this distinction matters because preventive treatment is recommended according to infection status and epidemiological risk, not because current tools can reliably determine whether infection is recent *MTB* infection, *MTB* infection, or biologically active (Organization WH, 2024; Organization WH, 2020).

At the population level, *MTB* infection remains common, but only a minority of infected individuals will progress to active disease (Cohen et al., 2019). Epidemiological studies have shown that recent exposure carries a particularly high short-term risk, with the excess risk diminishing over time (Behr et al., 2018; Sloot et al., 2014). This pattern is also seen in pediatrics and birth-cohort studies, where incident infection and early conversion are linked to a greater likelihood of subsequent disease (da Costa et al., 2024). These observations support the idea that infection stage itself matters, and that the early post-exposure period may represent a biologically vulnerable window. Distinguishing recent *MTB* infection from remote *MTB* infection is clinically important because recent infection represents an early post-exposure period with a higher short-term risk of progression to active TB, whereas remote infection may reflect a more stable immune-containment state. However, current infection tests mainly detect immune sensitization and cannot reliably determine infection timing.

Current tests for *MTB* infection, including the tuberculin skin test (TST) and interferon-*γ* release assays (IGRAs), mainly detect immunological sensitization and have only modest prognostic value for future TB (Abubakar et al., 2018; Jonas et al., 2023). Newer antigen-based skin tests have improved infection detection, but they still do not address the timing or biological activity of infection (Xu et al., 2022; Gupta et al., 2020). Regulatory guidance has therefore expanded the use of antigen-based skin tests, yet the central limitation remains: these assays identify infection, but they do not distinguish recent from *MTB* infection (Organization WH, 2022; Lu et al., 2023). In parallel, the WHO diagnostic framework continues to recommend symptom assessment, imaging, and microbiological testing to exclude active disease, rather than relying on a single immune readout (Organization WH, 2025).

The host response to *MTB* is shaped by a complex cytokine network that coordinates innate activation, adaptive immunity, granuloma formation, and immune regulation (Scriba et al., 2017; O'Garra et al., 2013). Within this network, IL-12 and IL-23 support Th1/Th17-related responses, IL-17A contributes to inflammatory recruitment, and chemokines such as CCL1 participate in immune-cell trafficking and regulation. At the tissue level, TB is increasingly understood as a compartmentalized disease, in which peripheral immune measurements may only partially reflect the biology occurring in the lung (Scriba et al., 2017; O'Garra et al., 2013). Consistent with this view, blood-based transcriptomic studies have shown that systemic signatures can distinguish active TB from controls and predict disease risk, but they also highlight the heterogeneity of the host response and the limitations of single-marker approaches (Berry et al., 2010; Sweeney et al., 2016).

Previous host-response biomarker studies have identified blood transcriptomic, proteomic, or cytokine signatures for distinguishing active TB from *MTB* infection, healthy controls, or other respiratory diseases. However, relatively few studies have specifically compared recent *MTB* infection, remote *MTB* infection, and active TB within the same analytical framework. In particular, the peripheral cytokine features associated with documented recent infection remain insufficiently characterized. Against this background, we profiled 12 circulating cytokines and chemokines across recent *MTB* infection, *MTB* infection, and active TB in a multicenter cohort from Jiangsu Province, China. Our aim was to characterize peripheral immune profiles across operationally defined recent *MTB* infection, remote infection, and active TB, and to evaluate their potential value for distinguishing TB infection state.

## Materials and methods

### Study design and setting

We conducted a multicenter observational biomarker study in Jiangsu Province, eastern China. The study drew on two complementary surveillance platforms: a school-based TB outbreak investigation system, which enabled serial screening of close contacts after identification of an index case, and a routine school-entry screening program for newly enrolled students. Active TB cases were recruited concurrently from designated TB hospitals within the same catchment area.

### Participants and group definitions

Participants were assigned *a priori* to three mutually exclusive groups: recent *MTB* infection, remote *MTB* infection, and active TB. Recent *MTB* infection was operationally defined as documented immunological conversion among close contacts of a bacteriologically confirmed index case during school-based TB outbreaks. To ensure diagnostic rigor, a two-step screening and confirmation strategy was employed. Contacts initially underwent baseline screening within 2 weeks of index case identification using the ESAT6-CFP10 (EC) skin test. Those with a confirmed negative baseline result were re-screened 8–12 weeks (approximately 2–3 months) later. Individuals demonstrating a negative-to-positive EC skin test conversion (induration ≥5 mm) immediately underwent confirmatory blood testing using an IGRA (specifically, QuantiFERON-TB Gold [QFT]). Only participants with definitively confirmed positive IGRA results were enrolled in the recent infection cohort. Blood samples for plasma cytokine profiling were collected concurrently with the confirmatory IGRA draw, within 2 weeks of the positive conversion signal.

Remote *MTB* infection was defined as a positive *MTB* infection screening result identified during routine school-entry screening in participants without documented close contact with a patient with active TB within the preceding 2 years, and in whom active TB was excluded by symptom review, clinical assessment, and imaging. Similar to the recent infection group, initial positive skin test screening results were strictly verified by confirmatory IGRA testing prior to study enrollment.

To prevent misclassification bias, active TB was rigorously excluded in both the recent and remote infection groups using standardized criteria uniformly applied across all multicenter sites. Clinically, all participants underwent a structured symptom screening; individuals reporting any TB-associated symptoms (cough ≥2 weeks, hemoptysis, unexplained fever, night sweats, or weight loss) were excluded. Radiologically, a posterior–anterior chest radiograph (CXR) was mandatory. If the CXR revealed abnormalities suggestive of active TB, or if clinical ambiguity existed, high-resolution computed tomography and microbiological tests (sputum smear/culture or Xpert MTB/RIF) were performed. Participants with any suspicious findings were excluded. These protocols strictly followed the Chinese National Health Commission Guidelines (WS 288–2017).

Active TB was defined according to Chinese national standards as bacteriologically confirmed or clinically diagnosed pulmonary TB (China NHCotPsRo, 2017).

To minimize confounding from underlying health conditions, we applied stringent exclusion criteria across all groups. We strictly excluded individuals with HIV infection, other immunodeficiency states, autoimmune diseases, malignancies, and severe systemic co-morbidities (such as uncontrolled diabetes mellitus or severe renal/hepatic dysfunction). Furthermore, individuals with any acute infection within the preceding 2 weeks, current immunosuppressive therapy, or a history of previous TB treatment were deemed ineligible. Consequently, the enrolled cohorts effectively represent an otherwise generally healthy population devoid of major co-morbidity burdens. All participants provided written informed consent.

### Biological sample collection and cytokine measurement

Peripheral venous blood was collected at enrolment and centrifuged at 10,000 rpm for 10 min. Plasma supernatants were stored at −80 °C until analysis. Concentrations of 12 cytokines and chemokines including IL-2, IL-4, IL-5, IL-8, IL-10, IL-12, IL-17A, IL-23, CCL1, CCL23, bFGF, and IL-1ra were measured using a Luminex-based multiplex immunoassay platform (Bio-Plex 200, Bio-Rad) with two commercial kits (MILLIPLEX Human High Sensitivity T Cell Panel, HSTCSF-28SK; Human Premixed Multi-Analyte Kit, LXSAHM-5), according to the manufacturer’s instructions. All samples were analyzed in duplicate. Prespecified quality thresholds were a coefficient of variation <20% and recovery between 70 and 130%. Cytokine concentrations were calculated using manufacturer-recommended calibration curves within the validated analytical ranges of each assay. The 12 cytokines and chemokines were selected *a priori* to represent major immune pathways implicated in TB, including Th1/Th17 immunity, immune regulation, chemokine-mediated cell recruitment, and tissue remodeling.

### Statistical analysis

Data distribution was assessed using the Shapiro–Wilk test, and homogeneity of variance was evaluated using Levene’s test. Because cytokine concentrations were non-normally distributed and several biomarkers showed heterogeneous variances, continuous variables are presented as median (interquartile range, IQR), and non-parametric statistical methods were used throughout the primary analyses.

Three-group comparisons were performed using the Kruskal-Wallis test followed by Dunn’s *post-hoc* test, whereas pairwise comparisons were performed using the Wilcoxon rank-sum test. Because age differed substantially between the infection groups and the active TB group, additional analyses were performed using age- and sex-adjusted residuals derived from linear regression models. To strictly control for multiple testing, a two-stage False Discovery Rate (FDR) correction (Benjamini-Hochberg procedure) was implemented. First, FDR correction was applied across the 12 omnibus tests. Second, for cytokines achieving an FDR-adjusted *p* < 0.05 in the omnibus test, a subsequent FDR correction was applied across the pairwise Dunn’s comparisons. Statistical significance is strictly defined based on these FDR-adjusted *p*-values.

Effect sizes were estimated using eta-squared (η^2^) for omnibus comparisons and rank-biserial correlation (r) for pairwise comparisons. Between-group differences were expressed as Log_2_ fold change (log_2_FC), with 95% confidence intervals estimated by bootstrap resampling (2,000 iterations).

Principal component analysis (PCA), t-distributed stochastic neighbor embedding (t-SNE), and uniform manifold approximation and projection (UMAP) were used to visualize overall cytokine patterns in an unsupervised manner.

Biomarker prioritization was performed using an ensemble feature-selection framework integrating LASSO regression, random forest, and XGBoost. Diagnostic models were evaluated using 100 repeated Monte Carlo cross-validation iterations with a stratified 7:3 train-test split to preserve class proportions. Hyperparameter tuning was performed using the training data only, and model performance was evaluated on the independent test set. Model performance was assessed using receiver operating characteristic (ROC) analysis, calibration plots, and decision curve analysis (DCA).

All statistical analyses were two-sided and performed using R software (version 4.2.1).

## Results

### Participant characteristics

**Figure 1:**
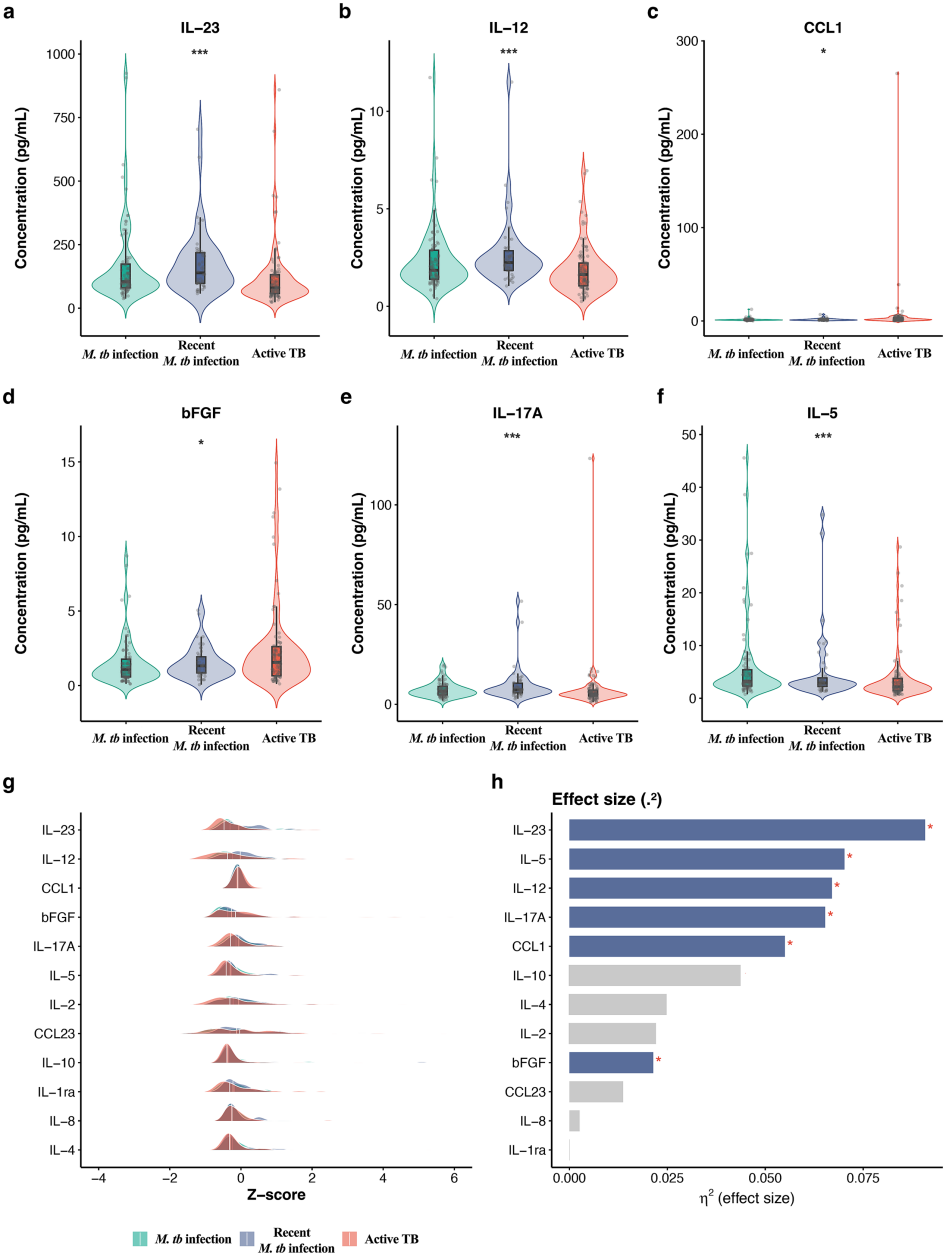
Plasma cytokine concentrations across *MTB* infection, recent *MTB* infection, and active tuberculosis. **(a)** Violin plots with embedded box plots showing IL-23 concentrations across the three groups; asterisks indicate Kruskal–Wallis significance after FDR correction (*p* < 0.01). **(b)** IL-12 concentrations across groups (*p* < 0.01). **(c)** CCL1 concentrations across groups (*p < 0.05). **(d)** bFGF concentrations across groups (unadjusted *p* = 0.040, FDR-adjusted *p* = 0.055). **(e)** IL-17A concentrations across groups (*p < 0.05). **(f)** IL-5 concentrations across groups (*p < 0.05). **(g)** Ridge plots of z-scored biomarker concentrations for all 12 analytes stratified by group, ordered by effect size. **(h)** Bar chart of η^2^ effect sizes from Kruskal–Wallis omnibus tests for all 12 biomarkers; red asterisks denote FDR-adjusted *p* < 0.05; blue bars indicate significance and gray bars indicate non-significance.

The study ultimately enrolled a total of 228 participants: 88 individuals (38.6%) with *MTB* infection, 44 (19.3%) with recent infection, and 96 (42.1%) diagnosed with active TB. While the sex distribution remained comparable across these clinical categories (Table 1; χ^2^ = 2.09, *p* = 0.351), we noted a significant age disparity (H = 101.4, *p* < 0.001). Specifically, both infection cohorts were younger, sharing a median age of 18.0 years, whereas the active TB group was notably older with a median of 27.0 years. The overall distribution of plasma cytokine concentrations for the entire study population is visualized in Figure 1. To support the reliability of group classification, we further clarified the tuberculosis-related testing results. All participants with recent *MTB* infection had documented EC skin test conversion and confirmatory QFT positivity. All participants with remote *MTB* infection had positive infection screening results confirmed by QuantiFERON-TB Gold testing, without documented close contact within the preceding 2 years. Active TB was excluded from both infection groups by standardized symptom screening and imaging, and all active TB patients included in this study were microbiologically confirmed.

**Table 1:** Baseline characteristics and plasma cytokine concentrations of the study population.

Characteristic	Overall *N* = 228^a^	*MTB* infection *N* = 88^a^	Recent *MTB* infection *N* = 44^a^	Active TB *N* = 96^a^	*p*-value^b^	FDR *P*^c^
Sex, n (%)					0.351	0.4
Female	101 (44%)	35 (40%)	18 (41%)	48 (50%)		
Male	127 (56%)	53 (60%)	26 (59%)	48 (50%)		
Age (years)	18.50 (16.50, 24.00)	18.00 (14.00, 20.00)	18.00 (17.50, 18.00)	27.00 (18.00, 54.50)	<0.001	<0.001
IL-10	7.31 (5.47, 16.17)	8.55 (6.04, 26.97)	7.33 (5.75, 14.27)	6.33 (4.43, 11.32)	0.003	0.005
IL-12	1.84 (1.32, 2.58)	1.85 (1.39, 2.88)	2.24 (1.84, 2.89)	1.63 (1.04, 2.23)	<0.001	<0.001
IL-17A	6.15 (4.58, 8.38)	6.50 (4.70, 9.03)	7.24 (5.69, 10.68)	5.12 (4.16, 7.12)	<0.001	<0.001
IL-2	1.75 (1.25, 2.44)	1.75 (1.35, 2.38)	2.01 (1.60, 2.72)	1.61 (1.13, 2.31)	0.031	0.048
IL-4	35.69 (26.18, 61.59)	41.82 (28.13, 75.96)	35.84 (26.34, 62.94)	33.05 (22.90, 47.62)	0.023	0.040
IL-23	100.86 (71.85, 164.94)	104.19 (79.95, 175.57)	138.98 (97.59, 219.02)	80.16 (57.62, 132.14)	<0.001	<0.001
IL-5	2.82 (1.86, 4.44)	3.19 (2.29, 5.44)	2.90 (2.15, 3.99)	2.17 (1.46, 3.76)	<0.001	<0.001
IL-8	45.60 (14.09, 114.64)	42.17 (11.44, 103.10)	45.36 (19.68, 104.83)	61.08 (15.62, 124.66)	0.300	0.300
CCL1	1.25 (0.93, 1.96)	1.23 (0.93, 1.56)	1.24 (0.85, 1.88)	1.61 (1.03, 3.25)	<0.001	0.002
bFGF	1.32 (0.62, 2.27)	1.08 (0.58, 1.77)	1.32 (0.85, 2.05)	1.56 (0.62, 2.63)	0.040	0.055
IL-1ra	778.52 (518.86, 1,178.33)	843.34 (525.79, 1,166.11)	793.92 (632.34, 1,151.17)	691.45 (437.64, 1,271.45)	0.400	0.400
CCL23	353.05 (252.54, 478.87)	322.65 (231.60, 416.80)	358.63 (286.79, 534.78)	376.09 (250.03, 515.96)	0.079	0.100

a n (%); Median (Q1, Q3).

b Pearson’s Chi-squared test; Kruskal-Wallis rank sum test.

c False discovery rate correction for multiple testing.

Values are presented as median (IQR) for continuous variables and n (%) for categorical variables. *p* values were calculated using the Kruskal–Wallis test for continuous variables and the χ^2^ test for categorical variables. FDR, false discovery rate. MTB, *Mycobacterium tuberculosis*; TB, tuberculosis.

### Plasma cytokine profiles across infection stages

In the initial unadjusted three-group comparison, eight of the 12 quantified analytes showed significant variation following FDR correction (Table 1): IL-10, IL-12, IL-17A, IL-2, IL-4, IL-23, IL-5, and CCL1. Interestingly, the recent infection group exhibited the highest median concentrations for IL-23 (138.98 pg./mL), IL-12 (2.24 pg./mL), and IL-17A (7.24 pg./mL). Conversely, the active TB cohort presented the lowest levels for these markers (80.16 pg./mL, 1.63 pg./mL, and 5.12 pg./mL, respectively; all FDR-adjusted *p* < 0.001). We observed a similar trajectory for IL-2, which peaked in the recent infection group (2.01 pg./mL) and reached its minimum in active TB (1.61 pg./mL), though with a distinct significance level (FDR-adjusted *p* = 0.048). *MTB* infection was instead associated with the highest median levels of IL-10 (8.55 pg./mL) and IL-5 (3.19 pg./mL). Detailed results of the pairwise comparisons between these groups are provided in Supplementary Table 1.

To account for the substantial age difference between groups, we subsequently performed age- and sex-adjusted analyses. Following covariate adjustment and independent FDR correction of the adjusted *p* values, six analytes remained statistically significant (Table 2). Following covariate adjustment and independent FDR correction, six analytes remained statistically significant (Table 2), whereas IL-2 and the remaining biomarkers no longer reached significance.

**Table 2:** Age- and sex-adjusted comparisons of plasma cytokines across recent *MTB* infection, *MTB* infection, and active tuberculosis.

Biomarker	Remote *MTB* infection	Recent *MTB* infection	Active TB	F statistic	*p* value	FDR *P*	η^2^
IL-23	104.19 (80.26, 173.55)	138.98 (97.94, 218.33)	80.16 (57.82, 131.70)	8.032	0.0004	0.0051	0.0908
IL-12	1.85 (1.39, 2.88)	2.24 (1.84, 2.85)	1.63 (1.05, 2.22)	7.028	0.0011	0.0066	0.0670
CCL1	1.23 (0.93, 1.48)	1.24 (0.85, 1.83)	1.61 (1.03, 3.22)	4.612	0.0109	0.0327	0.0550
bFGF	1.08 (0.58, 1.77)	1.32 (0.85, 1.93)	1.56 (0.65, 2.61)	4.855	0.0087	0.0327	0.0214
IL-17A	6.50 (4.70, 8.98)	7.23 (5.77, 10.59)	5.11 (4.18, 7.06)	3.952	0.0206	0.0411	0.0653
IL-5	3.19 (2.31, 5.41)	2.90 (2.22, 3.87)	2.17 (1.46, 3.74)	3.958	0.0205	0.0411	0.0702
IL-2	1.75 (1.35, 2.38)	2.00 (1.60, 2.69)	1.61 (1.14, 2.25)	2.358	0.0970	0.1454	0.0221
CCL23	322.64 (233.16, 416.31)	358.63 (286.79, 518.57)	376.09 (250.03, 513.12)	2.467	0.0871	0.1454	0.0137
IL-10	8.54 (6.06, 26.79)	7.33 (5.75, 13.59)	6.33 (4.43, 11.22)	1.742	0.1776	0.2238	0.0437
IL-1ra	843.34 (527.30, 1161.30)	793.92 (633.04, 1146.21)	691.44 (441.06, 1270.21)	1.692	0.1865	0.2238	0.0001
IL-8	42.17 (11.52, 101.81)	45.36 (20.38, 101.26)	61.08 (15.64, 123.48)	0.929	0.3966	0.4327	0.0026
IL-4	41.81 (28.13, 74.38)	35.84 (26.42, 62.25)	33.05 (22.90, 47.58)	0.566	0.5686	0.5686	0.0248

MTB, Mycobacterium tuberculosis; TB, tuberculosis; FDR, false discovery rate. F statistics were derived from age- and sex-adjusted residual models. η^2^, eta-squared effect size; FDR, false discovery rate.

### Combined MTB infection vs. active TB

To better understand the transition from infection to disease, we evaluated differences between the combined infection group (*n* = 132) and the active TB group (*n* = 96). Following FDR correction, five analytes displayed significant differences (Table 3). Active TB was characterized by a marked elevation in IL-8 levels (Log₂FC = 0.457, FDR-adjusted *p* = 0.0134). In contrast, four markers, IL-12 (Log₂FC = −0.306), IL-1ra (Log₂FC = −0.238), IL-10 (Log₂FC = −0.354), and IL-23 (Log₂FC = −0.449), were significantly lower in the active disease cohort (all FDR-adjusted *p* < 0.05).

**Table 3:** Differential plasma cytokine expression between combined infection and active tuberculosis.

Biomarker	*MTB* infection [median(IQR)]	Active TB [median(IQR)]	Log₂FC	95%CI	FDR *p*	Effect r
IL-8	44.49 (13.77, 101.81)	61.08 (15.64, 123.48)	0.457	−0.484, 1.278	0.0134	0.216
IL-12	2.01 (1.50, 2.88)	1.63 (1.05, 2.22)	−0.306	−0.597, −0.138	0.0142	0.193
IL-1ra	815.66 (570.38, 1157.64)	691.44 (441.06, 1270.21)	−0.238	−0.487, 0.072	0.0142	0.198
IL-10	8.09 (5.99, 24.79)	6.33 (4.43, 11.22)	−0.354	−0.646, −0.023	0.0207	−0.174
IL-23	109.40 (84.73, 190.63)	80.16 (57.82, 131.70)	−0.449	−0.748, −0.177	0.0207	0.177
CCL1	1.24 (0.93, 1.61) [*n* = 131]	1.61 (1.03, 3.22) [*n* = 95]	0.377	0.012, 0.860	0.0586	−0.145
IL-4	40.31 (28.09, 72.27)	33.05 (22.90, 47.58)	−0.287	−0.597, 0.062	0.0654	−0.137
bFGF	1.18 (0.62, 1.81) [*n* = 121]	1.56 (0.65, 2.61) [*n* = 90]	0.403	0.086, 1.058	0.0895	−0.130
IL-2	1.80 (1.42, 2.48)	1.61 (1.14, 2.25)	−0.161	−0.471, 0.033	0.0998	0.118
IL-17A	6.71 (5.14, 9.21)	5.11 (4.18, 7.06)	−0.392	−0.573, −0.189	0.4160	0.054
IL-5	3.08 (2.29, 5.09)	2.17 (1.46, 3.74)	−0.506	−0.646, −0.213	0.4160	−0.056
CCL23	340.22 (258.73, 454.59) [*n* = 131]	376.09 (250.03, 513.12)	0.145	−0.191, 0.337	0.4160	−0.058

MTB, *Mycobacterium tuberculosis*; TB, tuberculosis. Log_2_FC, Log_2_ fold change (Active TB vs. LTBI); CI, confidence interval; Effect r, rank-biserial correlation; FDR, false discovery rate.

When assessing diagnostic utility through individual biomarkers, IL-8 demonstrated the highest discriminatory capacity for active TB vs. infection (AUC 0.636), followed by IL-10 (0.632), IL-1ra (0.613), and IL-12 (0.597; Table 4). However, no single biomarker achieved an AUC exceeding 0.70.

**Table 4:** Diagnostic performance of individual plasma cytokines for distinguishing active tuberculosis from infection.

Biomarker	AUC	95%CI	Sensitivity	Specificity
IL-8	0.636	0.556, 0.714	0.360	0.975
IL-10	0.632	0.548, 0.707	0.640	0.689
IL-1ra	0.613	0.531, 0.694	0.539	0.773
IL-12	0.597	0.516, 0.673	0.315	0.866
IL-4	0.593	0.518, 0.672	0.685	0.588
CCL1	0.593	0.515, 0.671	0.438	0.773
bFGF	0.586	0.504, 0.668	0.539	0.681
IL-23	0.585	0.509, 0.662	0.539	0.639
IL-5	0.571	0.492, 0.652	0.517	0.706
IL-2	0.560	0.479, 0.640	0.573	0.563
CCL23	0.549	0.469, 0.632	0.461	0.706
IL-17A	0.512	0.430, 0.592	0.685	0.387

AUC, Area under the Curve; CI: confidence interval. AUC, area under the receiver operating characteristic curve; CI, confidence interval; Sensitivity and specificity were calculated at the Youden index.

### Unsupervised analyses and inter-marker correlations

**Figure 2:**
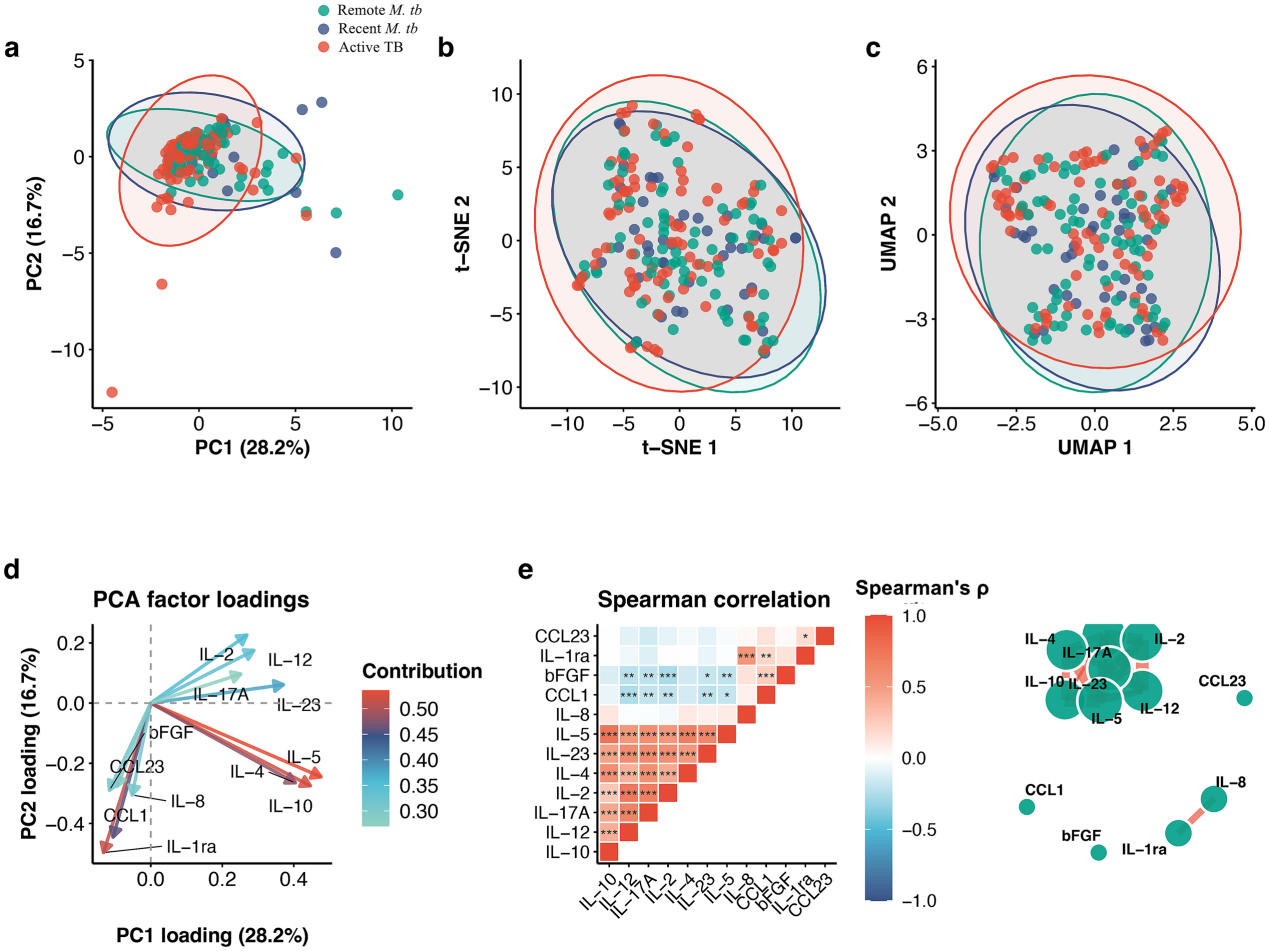
Unsupervised structure and inter-cytokine correlations in the full analysis. **(a–c)** Unsupervised clustering of participants with *MTB* infection (green), recent *MTB* infection (blue), and active TB (red) visualized using **(a)** principal component analysis (PCA) score plot, **(b)** t-distributed stochastic neighbor embedding (t-SNE), and **(c)** uniform manifold approximation and projection (UMAP). Ellipses represent 95% confidence intervals for each group. **(d)** PCA factor-loading biplot illustrating the contribution of individual cytokines to the first two principal components (PC1: 28.2%; PC2: 16.7%). Arrow length and color intensity reflect the proportional contribution to the total variance. **(e)** Left: Spearman rank-correlation heatmap for all 12 analytes; color scale represents the correlation coefficient (*ρ*) from −1.0 (blue) to 1.0 (red). FDR adjusted significance (*p < 0.05, **p < 0.01, ***p < 0.001). Right: Co-expression network constructed using a threshold of |*ρ*| > 0.40. Node size reflects degree centrality (the number of significant correlations), and edge color indicates correlation direction. Colors indicate study groups throughout panels **(a–c)**: Remote *MTB* (green), Recent *MTB* (blue), and Active TB (red).

Our unsupervised analysis revealed that PCA accounted for 44.9% of the total variance, with PC1 and PC2 contributing 28.2 and 16.7%, respectively. Score plots from PCA, t-SNE, and UMAP demonstrated that while the two infection groups overlapped considerably, participants with active TB occupied a partially separate immunological region (Figures 2a–c). Loading plots indicated that IL-12, IL-2, IL-17A, and IL-23 aligned with the primary PC1 direction, whereas CCL1, IL-1ra, and IL-8 loaded in the opposite direction (Figure 2d). Furthermore, Spearman correlation analysis identified a dense cluster of positive correlations among the Th1/Th17-related cytokines (Figure 2e; Supplementary Table 2).

### Feature selection and multivariable diagnostic models for active TB

**Figure 3:**
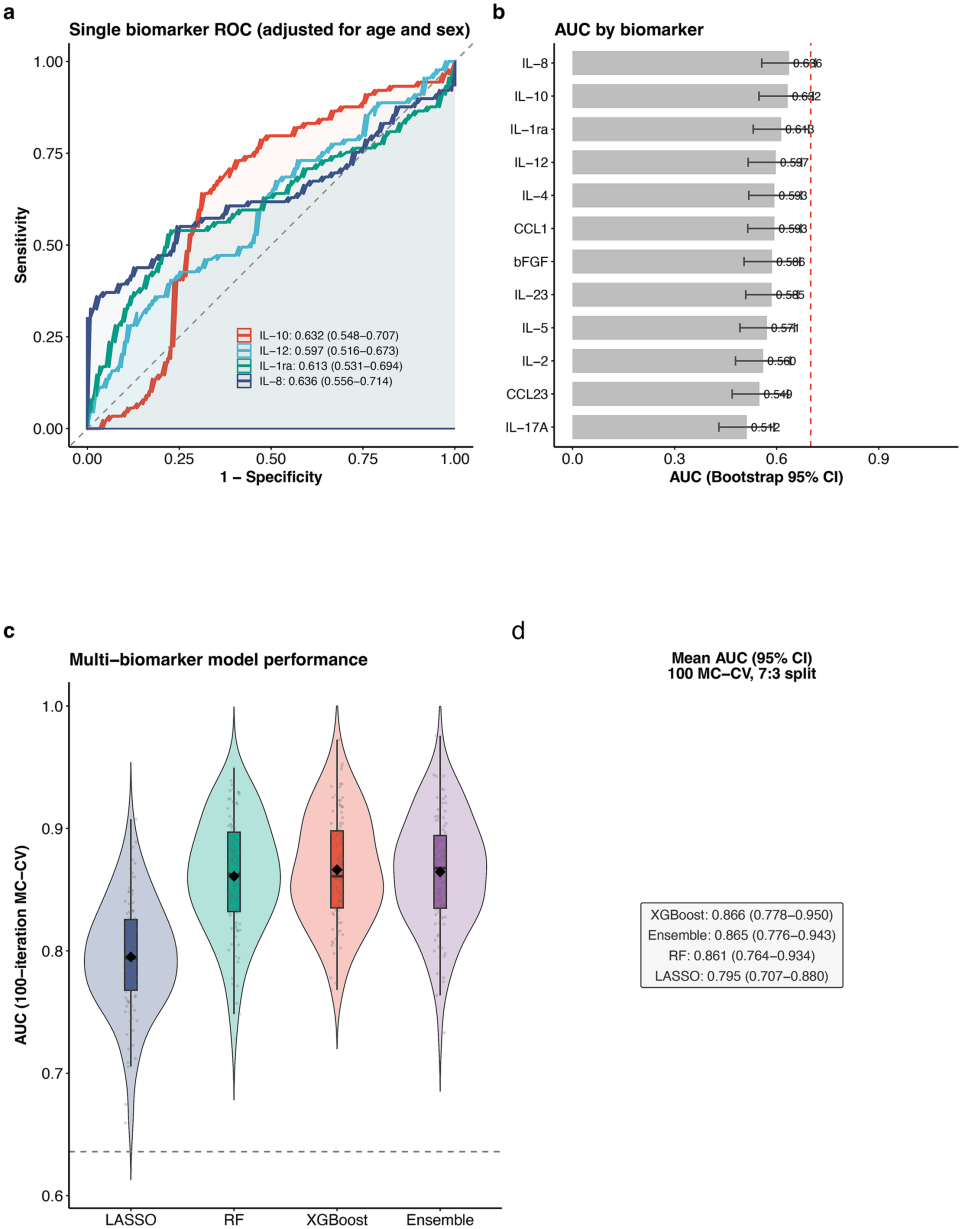
Ensemble feature selection for distinguishing active tuberculosis from infection. **(a)** LASSO stability selection frequencies across 100 repeated cross-validation runs; the red dashed line indicates the 0.50 threshold. **(b)** Random forest variable importance ranked by mean decrease in accuracy (ntree = 1,000). **(c)** XGBoost feature importance ranked by information gain. **(d)** Consensus dot plot integrating normalized importance scores from the three methods; the boxed area highlights the eight biomarkers selected for the final panel.

We prioritized biomarkers for diagnostic modeling using an ensemble feature-selection framework (Figure 3; Supplementary Table 3). LASSO stability selection, random forest, and XGBoost identified a consensus panel of eight biomarkers: CCL1, IL-23, IL-12, IL-1ra, bFGF, IL-8, IL-4, and CCL23 (Figures 3a–d).

**Figure 4:**
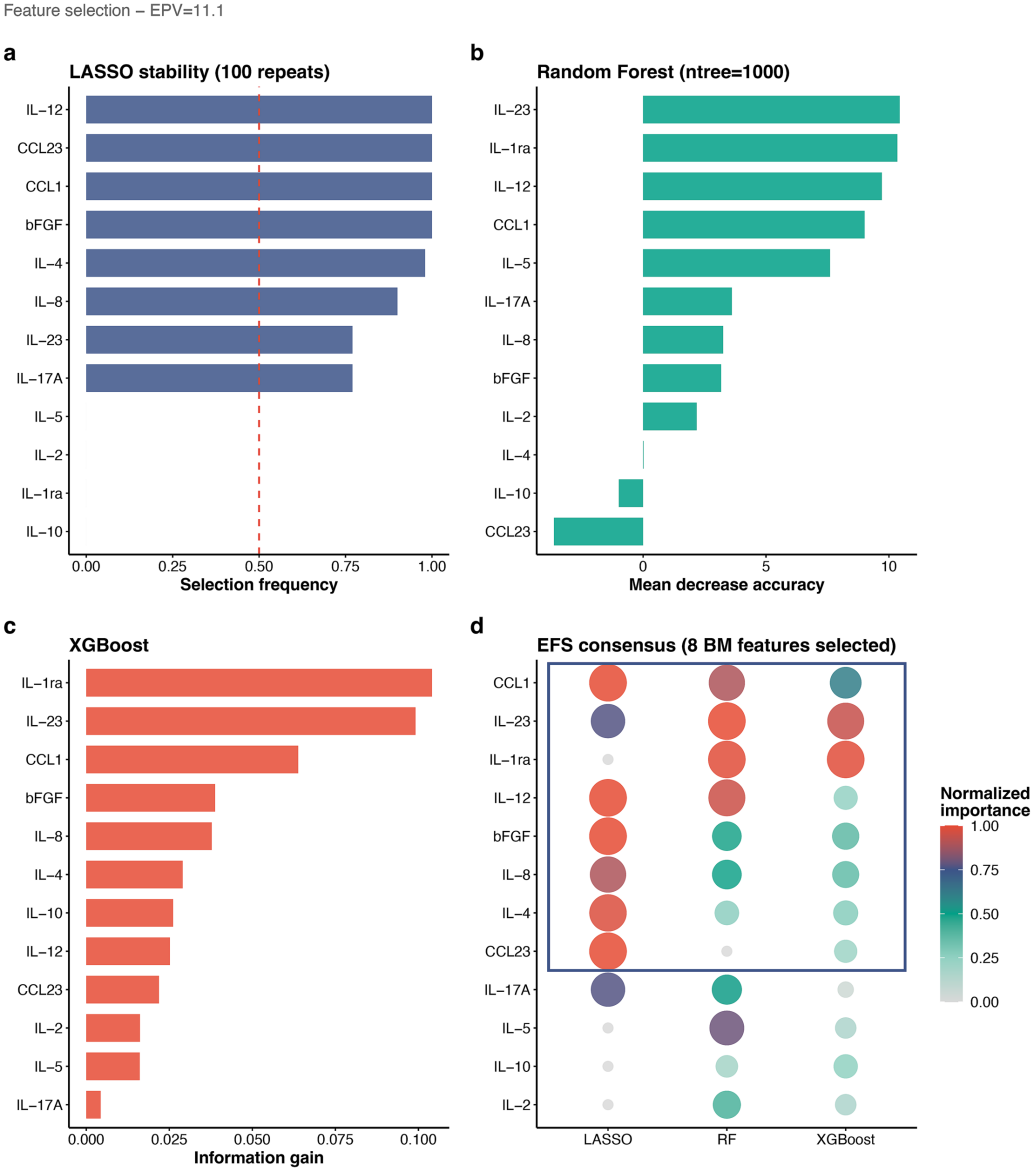
Diagnostic performance of individual and multivariable cytokine models for distinguishing active tuberculosis from infection. **(a)** ROC curves for the top single biomarkers after adjustment for age and sex. **(b)** AUC ranking of individual biomarkers with bootstrap 95% CIs. **(c)** AUC distributions across 100 Monte Carlo cross-validation iterations for the four multivariable models. **(d)** Mean AUC and 95% CIs across 100 cross-validation iterations.

The diagnostic performance of these markers was subsequently evaluated (Figure 4). While individual markers like IL-8 and IL-10 provided modest discrimination (Figures 4a,b), multivariable models yielded substantial improvements (Figure 4c). Across 100 iterations of Monte Carlo cross-validation, XGBoost achieved the highest mean AUC (0.866), followed by the ensemble model (0.865) and random forest (0.861; Figure 4d; Table 5). DCA indicated that the multivariable models, particularly the random forest model, provided higher net benefit than the treat-none strategy across a broad range of threshold probabilities, although the advantage over the treat-all strategy was modest (Supplementary Figure 1).

**Table 5:** Cross-validated performance of multivariable diagnostic models based on selected cytokines.

Model	Mean AUC	SD	95%CI	Valid iter
XGBoost	0.866	0.045	0.778, 0.950	100
Ensemble	0.865	0.046	0.776,0.943	100
RF	0.861	0.046	0.764, 0.934	100
LASSO	0.795	0.046	0.707, 0.880	100

AUC, Area under the Curve; CI, confidence interval. SD, Standard deviation. Values represent the mean AUC across 100 Monte Carlo cross-validation iterations. CI, confidence interval.

### Recent MTB infection vs. MTB infection subgroup analysis

A separate binary classification analysis was conducted to specifically decouple recent from *MTB* infection. After age- and sex-adjustment, IL-12 and IL-23 differed significantly between recent and *MTB* infection, whereas IL-2 did not. To overcome the limitations of single-cytokine comparisons, an ensemble feature-selection procedure was employed, which prioritized CCL23, IL-8, IL-12, and CCL1 as the primary multivariable indicators of infection timing (Supplementary Table 4). When evaluated as standalone markers, their discriminatory performance was modest; IL-12 demonstrated the highest AUC at 0.632, followed by CCL23 (0.629) and IL-23 (0.621) (Supplementary Table 5). However, the integration of these markers into machine learning models significantly enhanced the classification accuracy. Across 100 iterations of Monte Carlo cross-validation, the random forest model achieved the best performance with a mean AUC of 0.933 (95% CI: 0.831, 0.995), notably outperforming the ensemble (0.882) and XGBoost (0.705) models (Supplementary Table 6).

To facilitate interpretation of the subgroup analyses, *post-hoc* standardized effect size and statistical power analyses were performed (Supplementary Table 7). The observed differences between recent and remote infection were generally associated with small effect sizes, supporting cautious interpretation of these findings.

## Discussion

In this multicenter study, we identified distinct peripheral cytokine profiles across operationally defined recent *MTB* infection, remote infection, and active TB. The principal finding was not a generalized increase in systemic inflammation during active disease, but rather a shift in the overall peripheral immune profile. Active TB was characterized by lower circulating IL-12, IL-23, and IL-17A together with higher CCL1, suggesting a shift in peripheral immune composition rather than a uniform amplification or suppression of all immune mediators.

The reduced circulating IL-12, IL-23, and IL-17A concentrations observed in active TB may initially appear inconsistent with their established roles in antimycobacterial immunity (Scriba et al., 2017; O'Garra et al., 2013). However, TB is increasingly recognized as a compartmentalized disease, in which immune responses in peripheral blood do not necessarily reflect those occurring within pulmonary lesions. During active disease, antigen-specific effector cells and their associated cytokines may preferentially accumulate at the site of infection, resulting in relatively lower circulating concentrations despite ongoing local immune activation (Scriba et al., 2017; O'Garra et al., 2013). Persistent antigen stimulation may further contribute to immune dysregulation and altered cytokine production. This interpretation is consistent with blood transcriptomic studies showing that active TB is characterized by inflammatory and neutrophil-associated signatures rather than a simple amplification of protective T-cell responses (Berry et al., 2010; Sweeney et al., 2016). Nevertheless, because our study measured cytokines only in peripheral blood, these biological interpretations remain inferential.

Notably, the elevation of CCL1 should be interpreted within a different immunological context from the reduction in Th1/Th17-associated cytokines (Slight and Khader, 2013). CCL1 is a CCR8 ligand produced by activated monocytes/macrophages, dendritic cells, and T lymphocytes, and is primarily involved in leukocyte recruitment and immune regulation rather than directly reflecting protective Th1 immunity. Therefore, increased circulating CCL1 is not necessarily inconsistent with attenuated peripheral IL-12/IL-23 signaling. Instead, it may reflect enhanced inflammatory cell trafficking and immune remodeling accompanying active disease. Previous experimental studies have implicated the CCL1-CCR8 axis in the recruitment of regulatory and myeloid immune cells, which may contribute to the altered inflammatory milieu within tuberculosis lesions (Barsheshet et al., 2017; Tiffany et al., 1997). Because our study was cross-sectional and measured cytokines only in peripheral blood, these findings should not be interpreted as evidence that elevated CCL1 drives disease progression. Rather, CCL1 may represent a peripheral correlate of disease-associated immune dysregulation that warrants investigation in longitudinal and mechanistic studies.

The comparison between recent *MTB* infection and remote *MTB* infection provides additional insight into early host responses following *MTB* infection. Participants with operationally defined recent infection showed relatively higher circulating IL-12 and IL-23 concentrations than those with remote infection (Coussens et al., 2024), consistent with epidemiological observations that the risk of progression is greatest shortly after infection (Hamada et al., 2023; Behr et al., 2018; Sloot et al., 2014). However, the observed differences were modest, with small standardized effect sizes, indicating that the biological distinction between these two groups is subtle. Consequently, these findings should be regarded as hypothesis-generating rather than evidence of a discrete biological stage. Longitudinal studies incorporating serial immune measurements will be required to determine whether these peripheral cytokine patterns are associated with subsequent progression to active TB (Netea et al., 2016).

Previous TB biomarker studies have primarily focused on active TB diagnosis or progression prediction, whereas cytokine differences between recent and remote *MTB* infection remain less well characterized. By comparing documented recent infection, remote infection, and active TB within the same cohort, our study extends previous work by exploring peripheral immune differences related to both infection timing and disease activity. However, these findings remain exploratory and require external validation.

From a translational standpoint, the limited performance of individual cytokines was expected, reflecting the dynamic and multifactorial nature of host immune responses. IL-8 showed the highest individual discriminatory ability, although no single biomarker achieved an AUC greater than 0.70. In contrast, multivariable models integrating multiple cytokines substantially improved discrimination, consistent with previous studies demonstrating the superiority of multi-analyte signatures over individual biomarkers (Berry et al., 2010; Sweeney et al., 2016; Penn-Nicholson et al., 2019). From a translational standpoint, the six candidate molecules identified in this study may have potential value as an exploratory host-response panel rather than as a stand-alone diagnostic test. Because these analytes reflect host immune responses rather than pathogen-specific signals, the proposed cytokine panel should not be considered a replacement for TST, IGRA, or antigen-based skin testing. Instead, if externally validated, it may serve as a complementary tool after *MTB* infection has been established, supporting immune profiling, risk stratification, and clinical follow-up. In practical use, individuals with cytokine profiles closer to recent infection or active TB could be prioritized for closer symptom assessment, imaging review, and longitudinal monitoring. However, because the present study was cross-sectional and lacked external validation, this panel cannot yet be considered a validated tool for predicting TB progression. Although DCA suggested potential clinical benefit across exploratory threshold probabilities, no clinically validated operating threshold was identified, and therefore the present models should be regarded as hypothesis-generating.

Several limitations merit attention. First, this was a cross-sectional study, and therefore the observed peripheral cytokine profiles cannot establish temporal changes from recent infection to remote infection or active TB, nor can they determine whether these immune patterns are associated with subsequent disease progression. Longitudinal studies are required to address these questions. Second, participants were recruited through school-based outbreak investigations, routine school-entry screening, and designated TB hospitals in Jiangsu Province. Consequently, the study population consisted predominantly of adolescents and young adults with operationally defined infection, whereas active TB cases were older. Although all primary analyses were adjusted for age and sex, residual confounding and limited generalizability to older adults or populations with different TB epidemiology cannot be excluded. In addition, because only age and sex were systematically collected as baseline demographic variables, we were unable to evaluate the potential influence of other demographic or clinical characteristics on peripheral cytokine profiles. Third, only circulating cytokines were measured. Peripheral cytokine concentrations may not accurately reflect immune responses within pulmonary lesions, and the measured analytes represent host immune mediators rather than *Mycobacterium tuberculosis*-specific biomarkers. Moreover, the predefined cytokine panel captured only part of the host immune response and did not include other pathways, such as type I interferon signaling or inflammasome activation, that may contribute additional biological information. Finally, the subgroup differences between recent and remote infection were generally modest, and the study lacked external validation and non-TB disease controls. Therefore, the proposed cytokine signatures should be regarded as exploratory and require confirmation in larger, independent, and longitudinal cohorts before clinical application.

In summary, participants with operationally defined recent *MTB* infection exhibited peripheral cytokine profiles that differed modestly from those with remote infection and active TB. These findings support the concept that peripheral immune responses vary across tuberculosis infection states and provide a rationale for future longitudinal validation integrating cytokine profiling with broader multi-omic approaches (Anderson and Anderson, 2002; Palstrøm et al., 2022; Muneer et al., 2025; Scriba et al., 2021).

## Conclusion

Peripheral cytokine profiles differed across operationally defined recent *MTB* infection, remote infection, and active TB. Although the differences between recent and remote infection were modest, these findings suggest that peripheral immune responses vary across *MTB* infection states. Further longitudinal studies with external validation are required to determine their biological significance and potential clinical utility.

## Data Availability

The original contributions presented in the study are included in the article/Supplementary material, further inquiries can be directed to the corresponding authors.

## Supplementary material

SUPPLEMENTARY FIGURE 1 Decision curve analysis of the multivariable models. Net benefit was evaluated across threshold probabilities ranging from 0 to 0.95. The random forest model demonstrated the greatest overall net benefit, whereas all models should be interpreted as exploratory because no clinically validated decision threshold was predefined.
