# Supplementary material for: Peripheral cytokine distinguish recent from Mycobacterium tuberculosis infection and active tuberculosis
Source: Front Microbiol. 2026 Aug 5;17:1865147. doi: 10.3389/fmicb.2026.1865147 (PMC13486253; doi:10.3389/fmicb.2026.1865147)
Supplement: Supplementary file 2 [file Table_1.DOCX]

| **Supplementary Table 1. Complete pairwise Dunn post-hoc comparison results for all 12 biomarkers across the three groups (active TB, recent *M. tb* infection, *M. tb* infection).** | | | | | |
| --- | --- | --- | --- | --- | --- |
| **Biomarker** | **Comparison** | **Z stat** | **P raw** | **FDR *P*** | **Effect r** |
| IL-10 | Active TB - Recent *M. tb* infection | 2.703 | 0.0034 | 0.0103 | 0.179 |
| IL-10 | Active TB - *M. tb* infection | 1.915 | 0.0278 | 0.0416 | 0.127 |
| IL-10 | Recent *M. tb* infection - *M. tb* infection | -1.135 | 0.1283 | 0.1283 | 0.075 |
| IL-12 | Active TB - Recent *M. tb* infection | -3.496 | 0.0002 | 0.0007 | 0.232 |
| IL-12 | Active TB - *M. tb* infection | -1.820 | 0.0343 | 0.0343 | 0.121 |
| IL-12 | Recent *M. tb* infection - *M. tb* infection | 1.993 | 0.0232 | 0.0347 | 0.132 |
| IL-17A | Active TB - Recent *M. tb* infection | -1.801 | 0.0359 | 0.1076 | 0.119 |
| IL-17A | Active TB - *M. tb* infection | 0.000 | 0.4998 | 0.4998 | 0.000 |
| IL-17A | Recent *M. tb* infection - *M. tb* infection | 1.776 | 0.0379 | 0.0568 | 0.118 |
| IL-2 | Active TB - Recent *M. tb* infection | -2.499 | 0.0062 | 0.0187 | 0.165 |
| IL-2 | Active TB - *M. tb* infection | -0.889 | 0.1870 | 0.1870 | 0.059 |
| IL-2 | Recent *M. tb* infection - *M. tb* infection | 1.753 | 0.0398 | 0.0597 | 0.116 |
| IL-4 | Active TB - Recent *M. tb* infection | 1.977 | 0.0240 | 0.0721 | 0.131 |
| IL-4 | Active TB - *M. tb* infection | 1.609 | 0.0539 | 0.0808 | 0.107 |
| IL-4 | Recent *M. tb* infection - *M. tb* infection | -0.663 | 0.2535 | 0.2535 | 0.044 |
| IL-23 | Active TB - Recent *M. tb* infection | -3.282 | 0.0005 | 0.0015 | 0.217 |
| IL-23 | Active TB - *M. tb* infection | -1.625 | 0.0521 | 0.0521 | 0.108 |
| IL-23 | Recent *M. tb* infection - *M. tb* infection | 1.937 | 0.0263 | 0.0395 | 0.128 |
| IL-5 | Active TB - Recent *M. tb* infection | 1.166 | 0.1217 | 0.3652 | 0.077 |
| IL-5 | Active TB - *M. tb* infection | 0.444 | 0.3287 | 0.3287 | 0.029 |
| IL-5 | Recent *M. tb* infection - *M. tb* infection | -0.795 | 0.2132 | 0.3198 | 0.053 |
| IL-8 | Active TB - Recent *M. tb* infection | -2.282 | 0.0112 | 0.0169 | 0.151 |
| IL-8 | Active TB - *M. tb* infection | -3.037 | 0.0012 | 0.0036 | 0.201 |
| IL-8 | Recent *M. tb* infection - *M. tb* infection | -0.177 | 0.4296 | 0.4296 | 0.012 |
| CCL1 | Active TB - Recent *M. tb* infection | 1.584 | 0.0566 | 0.0849 | 0.105 |
| CCL1 | Active TB - *M. tb* infection | 1.997 | 0.0229 | 0.0687 | 0.133 |
| CCL1 | Recent *M. tb* infection - *M. tb* infection | 0.041 | 0.4838 | 0.4838 | 0.003 |
| bFGF | Active TB - Recent *M. tb* infection | 0.897 | 0.1850 | 0.2774 | 0.062 |
| bFGF | Active TB - *M. tb* infection | 2.038 | 0.0208 | 0.0623 | 0.140 |
| bFGF | Recent *M. tb* infection - *M. tb* infection | 0.785 | 0.2163 | 0.2163 | 0.054 |
| IL-1ra | Active TB - Recent *M. tb* infection | -2.364 | 0.0090 | 0.0136 | 0.157 |
| IL-1ra | Active TB - *M. tb* infection | -2.624 | 0.0043 | 0.0130 | 0.174 |
| IL-1ra | Recent *M. tb* infection - *M. tb* infection | 0.233 | 0.4080 | 0.4080 | 0.015 |
| CCL23 | Active TB - Recent *M. tb* infection | -0.749 | 0.2268 | 0.2268 | 0.050 |
| CCL23 | Active TB - *M. tb* infection | 1.642 | 0.0503 | 0.0755 | 0.109 |
| CCL23 | Recent *M. tb* infection - *M. tb* infection | 2.041 | 0.0206 | 0.0618 | 0.135 |
