# Supplementary material for: Peripheral cytokine distinguish recent from Mycobacterium tuberculosis infection and active tuberculosis
Source: Front Microbiol. 2026 Aug 5;17:1865147. doi: 10.3389/fmicb.2026.1865147 (PMC13486253; doi:10.3389/fmicb.2026.1865147)
Supplement: Supplementary file 3 [file Table_2.DOCX]

| **Supplementary Table 2. Spearman rank-correlation matrix for the 12 plasma cytokines and chemokines across the full cohort (n=228).** | | | | | | |
| --- | --- | --- | --- | --- | --- | --- |
| **Biomarker** | **Recent *M. tb* infection** | ***M. tb* infection** | **Log₂FC** | **95%CI** | **FDR *P*** | **Effect r** |
| IL-12 | 2.24 (1.84, 2.85) | 1.85 (1.39, 2.88) | 0.276 | 0.014, 0.513 | 0.1394 | 0.177 |
| IL-2 | 2.00 (1.60, 2.69) | 1.75 (1.35, 2.38) | 0.196 | -0.111, 0.474 | 0.1394 | 0.175 |
| IL-23 | 138.98 (97.94, 218.33) | 104.19 (80.26, 173.55) | 0.416 | -0.055, 0.850 | 0.1394 | 0.174 |
| CCL23 | 358.63 (286.79, 518.57) | 322.64 (233.16, 416.31) | 0.153 | -0.126, 0.427 | 0.1394 | 0.190 |
| IL-17A | 7.23 (5.77, 10.59) | 6.50 (4.70, 8.98) | 0.155 | -0.116, 0.410 | 0.1590 | 0.160 |
| IL-10 | 7.33 (5.75, 13.59) | 8.54 (6.06, 26.79) | -0.222 | -0.758, 0.206 | 0.5386 | -0.088 |
| IL-4 | 35.84 (26.42, 62.25) | 41.81 (28.13, 74.38) | -0.222 | -0.779, 0.276 | 0.5386 | -0.090 |
| bFGF | 1.32 (0.85, 1.93) | 1.08 (0.58, 1.77) | 0.290 | -0.473, 0.746 | 0.8001 | 0.057 |
| IL-5 | 2.90 (2.22, 3.87) | 3.19 (2.31, 5.41) | -0.138 | -0.413, 0.076 | 0.8645 | -0.038 |
| IL-8 | 45.36 (20.38, 101.26) | 42.17 (11.52, 101.81) | 0.105 | -0.814, 1.202 | 0.8645 | 0.023 |
| CCL1 | 1.24 (0.85, 1.83) | 1.23 (0.93, 1.48) | 0.012 | -0.556, 0.468 | 0.8645 | 0.023 |
| IL-1ra | 793.92 (633.04, 1146.21) | 843.34 (527.30, 1161.30) | -0.087 | -0.419, 0.321 | 0.9750 | 0.003 |
