# Supplementary material for: Peripheral cytokine distinguish recent from Mycobacterium tuberculosis infection and active tuberculosis
Source: Front Microbiol. 2026 Aug 5;17:1865147. doi: 10.3389/fmicb.2026.1865147 (PMC13486253; doi:10.3389/fmicb.2026.1865147)
Supplement: Supplementary file 4 [file Table_3.DOCX]

| **Supplementary Table 3. Ensemble feature-selection summary for distinguishing active tuberculosis from combined infection.** | | | | | |
| --- | --- | --- | --- | --- | --- |
| **Biomarker** | **LASSO freq** | **RF MDA** | **XGB Gain** | **Avg rank** | **Selected** |
| CCL1 | 1.00 | 9.0077 | 0.0638 | 3.1667 | Yes |
| IL-23 | 0.77 | 10.4383 | 0.0991 | 3.5000 | Yes |
| IL-12 | 1.00 | 9.7131 | 0.0252 | 4.5000 | Yes |
| IL-1ra | 0.00 | 10.3401 | 0.1041 | 4.5000 | Yes |
| bFGF | 1.00 | 3.1705 | 0.0388 | 4.8333 | Yes |
| IL-8 | 0.90 | 3.2569 | 0.0378 | 6.0000 | Yes |
| IL-4 | 0.98 | 0.0208 | 0.0290 | 7.0000 | Yes |
| CCL23 | 1.00 | -3.6252 | 0.0219 | 7.8333 | Yes |
| IL-17A | 0.77 | 3.6088 | 0.0043 | 8.5000 | No |
| IL-5 | 0.00 | 7.6042 | 0.0161 | 8.8333 | No |
| IL-10 | 0.00 | -0.9902 | 0.0261 | 9.5000 | No |
| IL-2 | 0.00 | 2.1790 | 0.0161 | 9.8333 | No |

LASSO stability selection frequency (100 repeats), random forest mean decrease in accuracy (RF MDA; ntree=1000), XGBoost information gain (XGB Gain), and average rank across the three methods are shown for each biomarker. Biomarkers with average rank ≤8.0 were selected for the final multivariable model.
