# Supplementary material for: Peripheral cytokine distinguish recent from Mycobacterium tuberculosis infection and active tuberculosis
Source: Front Microbiol. 2026 Aug 5;17:1865147. doi: 10.3389/fmicb.2026.1865147 (PMC13486253; doi:10.3389/fmicb.2026.1865147)
Supplement: Supplementary file 5 [file Table_4.DOCX]

| **Supplementary Table 4. Ensemble feature-selection summary for distinguishing recent from remote *Mycobacterium tuberculosis* infection.** | | | | | |
| --- | --- | --- | --- | --- | --- |
| **Biomarker** | **LASSO freq** | **RF MDA** | **XGB Gain** | **Avg rank** | **Selected** |
| CCL23 | 0.99 | 7.2978 | 0.0971 | 2.8333 | Yes |
| IL-8 | 0.36 | 15.9110 | 0.0738 | 3.1667 | Yes |
| IL-12 | 0.00 | 9.0258 | 0.1179 | 4.5000 | Yes |
| CCL1 | 0.00 | 10.3416 | 0.1078 | 4.5000 | Yes |
| IL-1ra | 0.69 | 4.4598 | 0.0131 | 5.6667 | No |
| IL-2 | 0.99 | 1.5669 | 0.0053 | 6.5000 | No |
| IL-23 | 0.00 | 4.2698 | 0.0376 | 6.8333 | No |
| bFGF | 0.00 | 3.9018 | 0.0145 | 8.1667 | No |
| IL-10 | 0.00 | 0.0232 | 0.0262 | 8.5000 | No |
| IL-17A | 0.01 | 0.1143 | 0.0000 | 8.8333 | No |
| IL-5 | 0.36 | -0.0214 | 0.0000 | 9.0000 | No |
| IL-4 | 0.00 | -2.1445 | 0.0261 | 9.5000 | No |
