# Supplementary material for: Peripheral cytokine distinguish recent from Mycobacterium tuberculosis infection and active tuberculosis
Source: Front Microbiol. 2026 Aug 5;17:1865147. doi: 10.3389/fmicb.2026.1865147 (PMC13486253; doi:10.3389/fmicb.2026.1865147)
Supplement: Supplementary file 6 [file Table_5.DOCX]

**Supplementary Table 5. Diagnostic performance of individual plasma cytokines for distinguishing recent infection from *Mycobacterium tuberculosis* infection.**

| **Biomarker** | **AUC** | **95%CI** | **Sensitivity** | **Specificity** |
| --- | --- | --- | --- | --- |
| IL-12 | 0.632 | 0.527, 0.737 | 0.571 | 0.701 |
| CCL23 | 0.629 | 0.526, 0.723 | 0.810 | 0.442 |
| IL-23 | 0.621 | 0.516, 0.722 | 0.667 | 0.597 |
| IL-2 | 0.617 | 0.513, 0.718 | 0.452 | 0.766 |
| IL-17A | 0.600 | 0.491, 0.706 | 0.762 | 0.468 |
| bFGF | 0.545 | 0.436, 0.649 | 0.786 | 0.390 |
| IL-4 | 0.541 | 0.433, 0.648 | 0.667 | 0.455 |
| IL-10 | 0.536 | 0.421, 0.646 | 0.310 | 0.831 |
| IL-8 | 0.509 | 0.395, 0.616 | 0.190 | 0.935 |
| CCL1 | 0.500 | 0.394, 0.609 | 0.405 | 0.779 |
| IL-5 | 0.492 | 0.384, 0.599 | 0.571 | 0.506 |
| IL-1ra | 0.476 | 0.376, 0.577 | 0.905 | 0.221 |
