# Supplementary material for: Peripheral cytokine distinguish recent from Mycobacterium tuberculosis infection and active tuberculosis
Source: Front Microbiol. 2026 Aug 5;17:1865147. doi: 10.3389/fmicb.2026.1865147 (PMC13486253; doi:10.3389/fmicb.2026.1865147)
Supplement: Supplementary file 7 [file Table_6.DOCX]

| **Supplementary Table 6. Cross-validated performance of multivariable models for distinguishing recent infection from *Mycobacterium* tuberculosis infection.** | | | | |
| --- | --- | --- | --- | --- |
| **Model** | **Mean AUC** | **SD** | **95%CI lower** | **95%CI upper** |
| RF | 0.933 | 0.041 | 0.831 | 0.995 |
| Ensemble | 0.882 | 0.067 | 0.740 | 0.975 |
| XGBoost | 0.705 | 0.106 | 0.449 | 0.872 |
| LASSO | 0.566 | 0.081 | 0.446 | 0.721 |
