# Supplementary material for: Peripheral cytokine distinguish recent from Mycobacterium tuberculosis infection and active tuberculosis
Source: Front Microbiol. 2026 Aug 5;17:1865147. doi: 10.3389/fmicb.2026.1865147 (PMC13486253; doi:10.3389/fmicb.2026.1865147)
Supplement: Supplementary file 8 [file Table_7.DOCX]

**Supplementary Table 7. Post-hoc power analyses were conducted to evaluate the robustness of subgroup comparisons.**

| **Comparison** | **Biomarker** | **n (group 1)** | **n (group 2)** | **Cohen's d** | **Effect grade** | **Power** | **Power (%)** | **n/group for 80%** | **Adequacy** |
| --- | --- | --- | --- | --- | --- | --- | --- | --- | --- |
| Combined *MTB* vs Active TB | bFGF | 121 | 90 | -0.410 | Small | 0.835 | 83.5% | 95 | Adequate |
| Combined *MTB* vs Active TB | IL-12 | 132 | 96 | 0.366 | Small | 0.775 | 77.5% | 119 | Marginal |
| Combined *MTB* vs Active TB | IL-23 | 132 | 96 | 0.338 | Small | 0.708 | 70.8% | 139 | Marginal |
| Combined *MTB* vs Active TB | IL-8 | 132 | 96 | -0.326 | Small | 0.678 | 67.8% | 149 | Marginal |
| Combined *MTB* vs Active TB | IL-4 | 132 | 96 | 0.296 | Small | 0.594 | 59.4% | 180 | Low |
| Combined *MTB* vs Active TB | IL-5 | 132 | 96 | 0.294 | Small | 0.589 | 58.9% | 183 | Low |
| Combined *MTB* vs Active TB | IL-10 | 132 | 96 | 0.287 | Small | 0.567 | 56.7% | 193 | Low |
| Combined *MTB* vs Active TB | CCL1 | 131 | 95 | -0.228 | Small | 0.391 | 39.1% | 304 | Very Low |
| Combined *MTB* vs Active TB | IL-2 | 132 | 96 | 0.171 | Negligible | 0.246 | 24.6% | 538 | Very Low |
| Combined *MTB* vs Active TB | CCL23 | 131 | 96 | -0.168 | Negligible | 0.238 | 23.8% | 558 | Very Low |
| Combined *MTB* vs Active TB | IL-1ra | 132 | 96 | -0.161 | Negligible | 0.224 | 22.4% | 605 | Very Low |
| Combined *MTB* vs Active TB | IL-17A | 132 | 96 | 0.101 | Negligible | 0.116 | 11.6% | 1,546 | Very Low |
| Recent vs Remote *MTB* | CCL23 | 43 | 88 | 0.482 | Small | 0.729 | 72.9% | 69 | Marginal |
| Recent vs Remote *MTB* | IL-2 | 44 | 88 | 0.417 | Small | 0.611 | 61.1% | 92 | Marginal |
| Recent vs Remote *MTB* | IL-17A | 44 | 88 | 0.383 | Small | 0.539 | 53.9% | 109 | Low |
| Recent vs Remote *MTB* | IL-12 | 44 | 88 | 0.208 | Small | 0.201 | 20.1% | 365 | Very Low |
| Recent vs Remote *MTB* | IL-1ra | 44 | 88 | -0.202 | Small | 0.192 | 19.2% | 388 | Very Low |
| Recent vs Remote *MTB* | IL-8 | 44 | 88 | 0.112 | Negligible | 0.092 | 9.2% | 1,255 | Very Low |
| Recent vs Remote *MTB* | IL-10 | 44 | 88 | -0.107 | Negligible | 0.089 | 8.9% | 1,365 | Very Low |
| Recent vs Remote *MTB* | IL-5 | 44 | 88 | -0.099 | Negligible | 0.083 | 8.3% | 1,593 | Very Low |
| Recent vs Remote *MTB* | IL-23 | 44 | 88 | 0.098 | Negligible | 0.082 | 8.2% | 1,638 | Very Low |
| Recent vs Remote *MTB* | IL-4 | 44 | 88 | -0.075 | Negligible | 0.069 | 6.9% | 2,827 | Very Low |
| Recent vs Remote *MTB* | CCL1 | 44 | 87 | 0.072 | Negligible | 0.067 | 6.7% | 3,006 | Very Low |
| Recent vs Remote *MTB* | bFGF | 43 | 78 | 0.003 | Negligible | 0.050 | 5% |  | Very Low |
| Power calculated using pwr.t2n.test(). alpha=0.05. n/group for 80%: required n per group (equal allocation). Green>=80%, Yellow 60-79%, Orange 40-59%, Red <40%. | | | | | | | | | |
